# Hepatitis B Virus (HBV) Treatment Eligibility in the UK: Retrospective Longitudinal Cohort Data to Explore the Impact of Changes in Clinical Guidelines

**DOI:** 10.1111/jvh.70098

**Published:** 2025-10-23

**Authors:** Cori Campbell, Tingyan Wang, Alexander J. Stockdale, Stacy Todd, Jakub Jaworski, Ben Glampson, Dimitri Papadimitriou, Erik Mayer, Hizni Salih, Gail Roadknight, Stephanie Little, Theresa Noble, Kinga A. Várnai, Cai Davis, Ashley I. Heinson, Michael George, Florina Borca, Timothy Roberts, Baptiste B. Ribeyre, Louise English, Leilei Zhu, NIHR HIC Viral Hepatitis and Liver Disease Consortium, Eleanor Barnes, Philippa C. Matthews, William Gelson, Graham S. Cooke, Salim Khakoo, Eleni Nastouli, Jim Davies, Kerrie Woods, Alexander J. Stockdale, Stephen Ryder, Ahmed M. Elsharkawy, Nicholas Easom, William Bernal, Shazaad Ahmad, Douglas Macdonald, Tingyan Wang, Cori Campbell, Simon Stanworth, Suzanne Maynard, Gail Roadknight, Stephanie Little, Kinga A. Várnai, Ben Glampson, Dimitri Papadimitriou, Luca Mercuri, Jakub Jaworski, Cai Davis, Florina Borca, Ashley Heinson, Michael George, Heidi MacNaughton, Yun Kim, Josune Olza Meneses, Louise English, Timothy Roberts, Baptiste Briot Ribeyre, Steve Harris, Stacy Todd, Lara Roberts, Javier Vilar, Ruth Norris, George Tilston, Ilina Serafimova, Sarah Montague, Juliette Verheyden, Irene Juurlink, Kathryn Jack, Alex Waldren‐Glenn, Lizzie Poole, Victoria Day, Berit Reglar, Kerrie Woods, Jim Davies, Graham S. Cooke, Eleni Nastouli, Salim I. Khakoo, William Gelson, Ahmed M. Elsharkawy, Eleanor Barnes, Philippa C. Matthews

**Affiliations:** ^1^ NIHR Oxford Biomedical Research Centre Oxford UK; ^2^ Nuffield Department of Medicine University of Oxford Oxford UK; ^3^ Department of Clinical Infection, Microbiology and Immunology, Institute of Infection, Veterinary and Ecological Sciences University of Liverpool Liverpool UK; ^4^ Tropical Infectious Diseases Unit, Royal Liverpool Hospital Liverpool University Hospitals NHS Trust Liverpool UK; ^5^ Cambridge University Hospitals NHS Foundation Trust Cambridge UK; ^6^ iCARE Secure Data Environment, NIHR Imperial Biomedical Research Centre Imperial College Healthcare NHS Trust London UK; ^7^ Faculty of Medicine, Department of Surgery and Cancer Imperial College London London UK; ^8^ NIHR Health Informatics Collaborative Oxford University Hospitals NHS Foundation Trust Oxford UK; ^9^ Southampton Emerging Therapies and Technologies Centre University Hospital Southampton NHS Foundation Trust Southampton UK; ^10^ Clinical Informatics Research Unit, Faculty of Medicine University of Southampton Southampton UK; ^11^ NIHR University College London Hospitals Biomedical Research Centre London UK; ^12^ Department of Computer Science University of Oxford Oxford UK; ^13^ Faculty of Medicine, Department of Infectious Disease Imperial College London London UK; ^14^ Department of Infection, Immunity and Inflammation UCL Great Ormond Street Institute of Child Health London UK; ^15^ Department of Virology UCLH London UK; ^16^ School of Clinical and Experimental Sciences, Faculty of Medicine University of Southampton Southampton UK; ^17^ Cambridge Liver Unit Cambridge University Hospitals NHS Foundation Trust Cambridge UK; ^18^ Liver Unit University Hospitals Birmingham Birmingham UK; ^19^ NIHR Biomedical Research Centre University Hospitals Birmingham Birmingham UK; ^20^ The Francis Crick Institute London UK; ^21^ Division of Infection and Immunity University College London London UK; ^22^ Department of Infectious Diseases University College London Hospital London UK

**Keywords:** criteria, eligibility, elimination, guidelines, HBV, hepatitis, NA therapy, tenofovir, treatment

## Abstract

Nucleos/tide analogue (NA) drugs are used for long‐term treatment of chronic hepatitis B virus (HBV) infection, with treatment eligibility criteria changing rapidly amidst globally evolving clinical guidelines. We aimed to quantify the prescription of NA drugs to date, and to undertake a preliminary assessment of the impact of relaxing treatment eligibility thresholds, leveraging a unique large real‐world secondary care dataset. We assimilated longitudinal clinical data, collected between February 1997 and April 2023 from adults with chronic HBV infection from six centres in England through the UK NIHR Health Informatics Collaborative (HIC) Viral Hepatitis and Liver Disease theme. We describe factors currently associated with the receipt of NA treatment and determine the proportion of the population who would become treatment eligible as thresholds change. Across 7558 adults with a mean follow‐up of 4.0 years (SD 3.9), NA treatment was prescribed in 2014/7558 (26.6%), and as expected according to guidelines at the time, was associated with HBV e‐antigen (HBeAg) positivity and alanine transferase (ALT) above the upper limit of normal (> ULN). Treatment was more likely in males, older adults, in Asian and Other ethnicities (compared to White), and less likely in socioeconomically deprived individuals. The proportion of treatment‐eligible individuals was 32.3% based on 2 records of ALT > ULN over 6–12 months, 41.7% based on ALT > ULN *and* viral load (VL) > 2000 IU/mL, and 95.1% based on detectable VL *and* either ALT > ULN *or* age > 30 years. Evolving clinical guidelines will lead to substantial increases in the proportion of individuals living with HBV who are eligible for treatment, underlining the need for services to adapt rapidly to the changing clinical environment.

## 1 Introduction

Chronic Hepatitis B (CHB) infection is a leading cause of morbidity from liver cancer and cirrhosis worldwide [1, 2], ranking as the first and third leading causes of death from these conditions, respectively [3, 4]. The World Health Organization's Global Health Sector Strategy (GHSS) on viral hepatitis identified key targets for the elimination of HBV as a public health threat by 2030. These include reducing incidence, morbidity, and mortality, and expanding antiviral coverage to individuals eligible for treatment [5]. GHSS targets include treatment for at least 80% of those who are eligible.

The mainstay of antiviral treatment is the administration of a nucleos/tide analogue (NA) agent, which suppresses HBV replication through reversible inhibition of the viral reverse transcriptase enzyme [6]. This reduces the long‐term risks of cirrhosis and HCC. NA treatment typically comprises a once‐daily oral dose of tenofovir disoproxil fumarate (TDF) or entecavir (ETV). However, NA agents are not curative and must be taken long‐term in most cases.

In 2022, the prevalence of HBV in England was estimated at 0.6% of the population, with regional variability (e.g., 1.5% in London) [7]. Screening is offered through a variety of services, including routine antenatal testing, opt‐out screening in selected Emergency Departments, and populations at high risk for exposure (including—but not limited to—people accessing sexual health services, people in prison or care facilities, people presenting with liver disease, migrants from medium/high endemicity countries, and those with other blood‐borne virus infections). However, there are recognised gaps in screening, and current estimates suggest that more than half of the population living with HBV have yet to be diagnosed.

In the UK (and most European countries), CHB is typically managed in specialist secondary or tertiary care clinics (led by hepatology, gastroenterology, infectious diseases, or sexual health services) [6, 8]. Laboratory investigations include HBV e‐antigen (HBeAg) status, HBV DNA viral load (VL) quantification, co‐infection status (HIV/HCV/HDV), and hepatic transaminases (such as alanine transferase, ALT) [6]. In some centres, HBV surface antigen (HBsAg) quantification is also now being adopted. Liver fibrosis can be assessed by transient elastography, ultrasound, and/or by calculation of laboratory‐based fibrosis scores. Therapy is currently reserved for those deemed at the highest risk of complications, based on algorithms that account for these variables, alongside age, sex, and family history. On this basis, a minority of people living with HBV have met criteria for antiviral treatment to date [6, 9, 10], but for any individual, disease markers and treatment eligibility may change over time, so regular surveillance is required.

In 2024, the WHO published a high‐profile report highlighting that the global public health response for HBV is not on track [11], and in parallel released new global guidelines, relaxing HBV treatment criteria with the intent of simplifying treatment roll‐out, reducing costs, and making treatment access more equitable, particularly for resource‐limited settings [12]. Likewise, some countries updated their national recommendations for HBV, moving in the same direction towards wider treatment, exemplified by China [13] and Brazil [14, 15]. Guidelines produced by the European Association for the Study of the Liver (EASL) followed in 2025 [16], and the American Association for the Study of Liver Disease (AASLD) is currently rewriting clinical practice guidelines.

This study used electronic health record (EHR) data within the dataset of the NIHR Health Informatics Collaborative (HIC) for Viral Hepatitis and Liver Disease theme [17], which collects demographic and longitudinal clinical data held by secondary and tertiary care centres. Our objectives were to assess the proportion of individuals in active care for HBV who have already been prescribed NA agents, to describe characteristics of this treated population and individual characteristics associated with antiviral treatment, and to determine the impact of changes in guidelines on the population who meet eligibility criteria for treatment.

## 2 Methods

### 2.1 NIHR Health Informatics Collaborative (HIC)

The HIC collects individual‐level anonymised longitudinal EHR data collected by the National Health Service (NHS), as previously described [17, 18]. Data elements are defined according to the standardised NHS Data Dictionary [19]. Data classes include demographics, laboratory tests, elastography scores, imaging, potential risk factors for liver disease, and information regarding antiviral treatment. Socioeconomic data were available for a subset of five NHS Trusts; deprivation was ascertained according to the Index of Multiple Deprivation (IMD) deciles, which were collapsed into quintiles for this analysis. The IMD is a multifactorial area‐level measure of deprivation incorporating crime, education, employment, income, and the living environment [20]. Definitions for ethnicity and sex/gender have been taken from the standardised NHS Data Dictionary (https://www.datadictionary.nhs.uk/), with ethnicity being self‐reported based on a standard NHS approach to data collection and defined according to census categories which are transferred into HIC coding. Duration of follow‐up varies by individual and by respective NHS site (due to variable clinical review and data collection processes).

Data storage and entry processes are unique to individual NHS sites, with different sites using a range of EHR interfaces (e.g., Cerner Millennium and Epic). Data collection processes have been detailed previously [17]. In brief, the HIC Viral Hepatitis and Liver Disease theme provides an informatics infrastructure via which data from all sites are harmonised and held on a central server, hosted by Oxford University Hospitals (OUH) NHS Foundation Trust. A comprehensive data governance framework has been developed to oversee data collection across the sites that use heterogeneous EHR processes and environments. Approved researchers access anonymised data remotely via a secure Trusted Research Environment.

Data from six centres was included in analyses for this study, based on availability of antiviral HBV treatment data at the time of the study. These were Cambridge University Hospitals NHS Foundation Trust [CUH], Imperial College Healthcare NHS Trust [ICHT], Liverpool University Hospitals NHS Foundation Trust [LUH], OUH; University College London Hospitals NHS Foundation Trust [UCLH], and University Hospital Southampton NHS Foundation Trust [UHS].

### 2.2 Ethics

The NIHR HIC Viral Hepatitis and Liver Disease theme database was approved by South Central—Oxford C Research Ethics Committee (REF Number: 21/SC/0060). The requirement for written informed consent is waived because data are anonymised before transmission to the central data repository, but individuals can choose not to have their data included via the National Data Opt‐out [21]. This specific project was approved by written submission to the theme scientific steering committee, which represents all participating centres.

### 2.3 Study Design and Follow‐Up

We retrospectively identified individuals with CHB, who were aged ≥ 18 years at the time of the earliest record of HBV diagnosis within the cohort dataset. Typically, CHB is defined as a persistence of HBsAg and/or HBV DNA for at least 6 months. However, as per terms agreed by the HIC viral hepatitis steering group, we used relaxed eligibility criteria, requiring only one positive HBsAg or DNA VL measurement, to avoid unnecessary exclusions. Data analysed here were collected between February 1997 and April 2023.

Treatment decisions are taken by clinicians in the centre leading care, and will typically be based on guidance issued by the National Institute for Health and Care Excellence (NICE) [6] or EASL recommendations. As these data are retrospective, individuals represented in this analysis will have initiated treatment prior to the change in WHO guidelines in 2024 and updated EASL guidelines in 2025 [10]. Prescribing decisions are also influenced by individual clinical judgement and patient choice. We could not reliably explore time to treatment initiation as the time of first prescription is not robustly recorded, and a proportion of individuals are already receiving treatment at cohort baseline if NA therapy was initiated prior to their data being represented in HIC.

### 2.4 Primary Aims

Our primary outcome of interest was antiviral treatment status (defined as a binary parameter; 0 = no treatment record at any point during the period of observation; 1 = treatment recorded at any time point throughout the period of observation). We investigated how demographics and laboratory measurements were associated with antiviral treatment, and evaluated the impact of different treatment criteria on the proportion of the whole CHB population becoming eligible for NA therapy under new thresholds.

### 2.5 Variable and Outcome Ascertainment

We considered different treatment scenarios, starting with the real‐world observation of those already prescribed NA therapy, and calculating the proportion of individuals with a record of treatment to date, using this as a baseline estimate of eligibility. We next applied revised treatment criteria based on new and emerging clinical guidelines (Table 1) to determine how these would increase the proportion of those eligible for treatment.

**TABLE 1:** Thresholds applied to determine treatment eligibility in people living with chronic HBV (CHB) infection.

Assessment applied	Criteria for treatment	Criteria applied to analysis of HIC dataset
Routine clinical assessment (UK setting)	Real world clinical evaluation in secondary care in England (clinicians apply NICE or EASL guidelines).	Observation of real world HBV cohort (Scenario (i))
WHO guidelines, 2024 [12]	In the absence of access to HBV DNA measurement, persistently abnormal ALT levels (defined as two ALT values > ULN^b^ during a 6–12‐month period), regardless of APRI score.	Two ALT values > ULN^b^ during a 6–12‐month period, regardless of other markers (Scenario (iii))
	Evidence of ‘significant fibrosis’ or cirrhosis (regardless of HBV DNA or ALT levels), based on APRI^a^ > 0.5 *or* transient elastography value of > 7 kPa *or* evidence of cirrhosis based on clinical criteria	APRI^a^ > 0.5, regardless of other markers (Scenario (iv)) Insufficient data for analysis of transient elastography or cirrhosis in this study.
	HBV DNA > 2000 IU/mL *and* ALT> ULN^b^ In adolescents ALT should be measured at least twice in a 6–12‐month period.	HBV DNA > 2000 IU/mL *and* ALT> ULN^b^ (Scenario (v)) Adolescents not evaluated in this study.
	Comorbidity, based on any of the following, (regardless of APRI, HBV DNA or ALT levels) Presence of coinfection (HIV, HCV and/or HDV) Family history of HCC or cirrhosis; Immune suppression; Extrahepatic manifestations of HBV infection.	APRI^a^ > 0.5 *or* coinfection with HIV/HCV/HDV (Scenario (vi)) Family history and other clinical history not available in this dataset.
	Detectable HBV DNA *and* either ALT> ULN *or* age > 30	Detectable HBV DNA *and* either ALT> ULN *or* age > 30 (Scenario (ix))
	‘Treat all’	HBsAg+ *or* HBV DNA detectable (Scenario (xi))
EASL guidelines, 2025 [16]	‘Persistently’ elevated ALT (even if HBV DNA < 2000 IU/mL)	Equivalent to WHO criteria (Scenario (iii))
	HBV DNA > 2000 IU/mL *and* any of the following ALT> ULN Transient elastography > 7 kPa Risk factors for HCC Low platelets Extra‐hepatic complications Immunosuppression Transmission risk	HBV DNA and ALT criteria equivalent to Scenario (v) Separately, evaluated low platelets^d^ as an alternative to ALT > ULN (Scenario (vii))
	Treat all, excluding the lowest risk group (those who do NOT need treatment, defined as HBeAg‐negative, persistent HBV DNA < 2000 IU/mL, persistently normal ALT, no signs of liver fibrosis) (i.e., fibroscan < 7 kPa)	DNA > 2000 iU/mL (at any timepoint) *or* ALT > ULN (at any timepoint) *or* HBeAg+ (at any timepoint) (Scenario (viii))
	‘Advanced fibrosis or cirrhosis’ defined as fibroscan > 8 kPa OR APRI > 0.5, with any level of detectable HBV DNA.	APRI > 0.5 *and* any positive HBV DNA
	All HBsAg‐positive individuals with detectable HBV DNA	HBV DNA positive at any time point
Chinese guidelines, [13]	HBV DNA detected *and* any of the following ALT > ULN^b^ Age > 30 years Family history of HBV‐related cirrhosis or HCC Histological or imaging assessment showing fibrosis Extra‐hepatic manifestations	HBV DNA detected and any of the following ALT > ULN^b^ *Or* age > 30 years
Brazilian guidelines [14, 15]	Compensated cirrhosis *and* HBsAg positive *and* detectable HBV DNA (regardless of other markers) Decompensated cirrhosis *and* HBsAg positive (regardless of other markers)	Not assessed because cirrhosis not clearly defined in this HIC dataset.
	For individuals in HBV ‘immunotolerant phase’^c^, if > 30 years *Or* family history of HCC/cirrhosis *Or* extrahepatic manifestations of HBV	Not assessed because no biopsy data, and no record of family history/cirrhosis/extrahepatic manifestations in this HIC dataset.
	In ‘chronic hepatitis’ phase, if ALT > ULN *And* HBV DNA > 2000–20,000 IU/mL Irrespective of HBeAg status	Corresponds to WHO guidelines (Scenario (v))

*Note:* Each criterion is mapped to a scenario, numbered from (i), which represents the population treated before guidelines were changed, through to (xi), being treat all. Scenarios are numbered in ranked order reflecting the impact on the proportion of the population eligible for treatment (presented in order in main text, Figure 2, Table S1).

Abbreviations: ALT, alanine transferase; HBV, hepatitis B virus; HCC, hepatocellular carcinoma; HIC, Health Informatics Collaborative; ULN, upper limit of normal; WHO, World Health Organization.

^a^ APRI—aspartate aminotransferase to platelet ratio index = [(AST/upper limit of normal) × 100/platelet count], with AST ULN = 40 U/L.

^b^ ULN for ALT defined by WHO guidelines as 30 U/L for males and 19 U/L for females.

^c^ Definition of immunotolerant infection from Brazilian guidelines: ‘HBeAg positivity, high HBV DNA (generally > 10^6^ or 10^7^ IU/mL), alanine aminotransferase (ALT) within ULN, and minimal or absent inflammation and fibrosis upon histopathology’.

^d^ Low platelets here defined as < 150 × 10^9^/L.

The widest treatment eligibility considered by the WHO provides flexibility for offering treatment to anyone who is confirmed to be HBsAg‐positive (in the absence of access to other laboratory testing and risk stratification) [12]. New EASL guidelines make similarly wide provision, although they anticipate that VL assessment should be accessible, stipulating that ‘in principle, all HBsAg‐positive individuals with detectable HBV DNA are candidates for antiviral therapy’ [16]. However, both WHO and EASL guidelines recognise that, in many cases, additional assessment tools and clinical assessment will guide a final treatment decision, and that personalised approaches are appropriate. Guidelines therefore recognise that while there are potential gains of adopting a ‘treat all’ approach, HBV infection is heterogeneous (including subgroups of individuals at very low risk of complications or transmission, who may not benefit from treatment), and that risks, feasibility, and costs of wider treatment need to be considered as part of decision‐making.

Treatment categories are based on thresholds included in WHO and/or EASL guidelines and others [12, 13, 14, 16], pending new guidelines from the other major liver societies (e.g., AASLD). ALT above the upper limit of normal (ULN) was defined as ≥ 30 U/L in males and ≥ 19 U/L in females as per WHO guidelines [12]. Aspartate transaminase (AST) ULN was defined as 40 U/L. A low platelet count was defined here as less than the lower limit of normal (150 × 10^9^/L). AST to Platelet Ratio Index (APRI) was calculated using the formula (AST Level (U/L)/AST ULN (U/L))/platelet count (×10^9^/L) ×100.

In each case, we assume the originally treated population continues established therapy, while others become newly eligible and are therefore added to the population treated at baseline. For untreated patients with multiple visits and clinical/laboratory measurements during follow‐up, all data points across the study period were evaluated against respective treatment eligibility criteria. On this basis, an individual meeting treatment criteria at any timepoint (based on the peak/nadir values during the whole period of follow‐up) was considered treatment eligible.

### 2.6 Statistical Analysis

We carried out analyses using R (version 4.1.0). Baseline characteristics were summarised for the whole study population using descriptive statistics. Means and standard deviations (SDs) or medians and interquartile ranges (IQRs) were presented for continuous measures. Patient counts and percentages were presented for categorical and binary variables.

We used univariable and multivariable logistic regression models to investigate factors associated with antiviral treatment. Odds ratios (ORs) and 95% confidence intervals (95% CIs) were reported for outputs. HBV VL and ALT were modelled as binary measures according to relevant thresholds (> 2000 IU/mL and > ULN, respectively). Factors were chosen for inclusion in the multivariable model by consideration of both the significance of univariable associations (where *p* < 0.1) together with biological/clinical relevance and previous literature. Logistic regression was used to investigate factors associated with antiviral treatment, without assuming a temporal association whereby factors would have been ascertained before treatment status was assigned. This was intentional, as the number of visits and therefore covariate measurements differs between individuals. Therefore, we could not make any temporality assumptions (ascertainment of time to either treatment initiation or censoring was not possible).

### 2.7 Patient and Public Involvement

Our clinical and research teams work in collaboration with patient representatives, who have been involved in ongoing and planning prospective HIC studies. We have met with patients and carers with lived experience of CHB from diverse backgrounds across various UK locations, including representation of those who take daily medication, and clinical monitoring. They are involved in discussing research priorities, methods, and design.

## 3 Results

### 3.1 Cohort Characteristics at Baseline

In total, 7558 adults living with CHB in England were eligible for inclusion in this analysis (baseline characteristics in Table 2). Mean follow‐up was 4.0 years (SD 3.9). Just under half the population was of self‐reported White ethnicity (*n* = 3414, 45.2%), compared to 12.1% (*n* = 917) Asian, 17.8% (*n* = 1342) Black, 2.6% (*n* = 197) mixed, and 22.3% ‘other’ ethnicities (*n* = 1688). IMD was available for 6891 (91.2%) of our study population, among whom over half belonged to the two most deprived UK population quintiles (most deprived IMD quintile [*n* = 1759, 25.5%] and second most deprived IMD quintile [1711, 24.8%]). A minority of individuals had documented coinfection with HIV (1.2%, *n* = 91), HCV (1.0%, *n* = 79), or HDV (1.0%, *n* = 75).

**TABLE 2:** Baseline characteristics of individuals included in the HIC analysis investigating factors associated with antiviral (NA) treatment in adults living with chronic HBV infection.

Characteristic	Total population	Untreated population	Treated population
*N* (% of total)	7558 (100)	5544 (73.4)	2014 (26.6)
Follow‐up time (years), mean (SD)	4.0 (3.9)	4.3 (4.1)	3.1 (3.12)
Age group, *n* (%)			
< 25 years	436 (5.8)	344 (6.2)	92 (4.6)
25–34 years	2119 (28.0)	1680 (30.3)	439 (21.8)
35–44 years	2206 (29.2)	1678 (30.3)	528 (26.2)
45–54 years	1398 (18.5)	931 (16.8)	467 (23.2)
55–64 years	855 (11.3)	542 (9.8)	313 (15.5)
≥ 65 years	543 (7.2)	368 (6.6)	175 (8.7)
Index of multiple deprivation (IMD) quintile, *n* (%)			
First quintile (most deprived)	1759 (25.5)	1348 (26.1)	411 (23.9)
Second quintile	1711 (24.8)	1304 (25.2)	407 (23.7)
Third quintile	1416 (20.5)	1043 (20.2)	373 (21.7)
Fourth quintile	1167 (16.9)	863 (16.7)	304 (17.7)
Fifth quintile (least deprived)	838 (12.2)	613 (11.9)	225 (13.1)
Missing	667 (8.8)	373 (6.7)	294 (14.6)
Sex, *n* (%)			
Female	3419 (45.2)	2689 (48.5)	730 (36.2)
Male	4137 (54.7)	2853 (51.5)	1284 (63.8)
Missing	< 5	< 5	0
Ethnicity (self‐reported), *n* (%)			
White	3414 (45.2)	2639 (47.6)	775 (38.5)
Asian	917 (12.1)	615 (11.1)	302 (15.0)
Black	1342 (17.8)	1019 (18.4)	323 (16.0)
Mixed	197 (2.6)	144 (2.6)	53 (2.6)
Other	1688 (22.3)	1127 (20.3)	561 (27.9)
Other clinical and laboratory parameters			
HIV coinfection, *n* (%)	91 (1.2)	51 (0.9)	40 (2.0)
HCV coinfection, *n* (%)	79 (1.0)	51 (0.9)	28 (1.4)
HDV coinfection, *n* (%)	75 (1.0)	46 (0.8)	29 (1.4)
ALT > ULN	4534 (60.0)	3188 (57.5)	1346 (66.8)
HBV VL > 2000 IU/mL	1597 (21.1)	1141 (20.6)	456 (22.6)

*Note:* Percentages are expressed out of the column total, as stated in the first row.

Abbreviations: ALT > ULN, upper limit of normal (≥ 30 IU/ML in males and ≥ 19 IU/ML in females); ALT, alanine aminotransferase; HBeAg, hepatitis B virus E antigen; HBsAg, hepatitis B virus surface antigen; HBV VL, hepatitis B virus viral load; HCV, hepatitis C virus; HDV, hepatitis Delta virus; HIV, human immunodeficiency virus; IQR, interquartile range.

### 3.2 Factors Associated With Receipt of Antiviral Treatment

**FIGURE 1:**
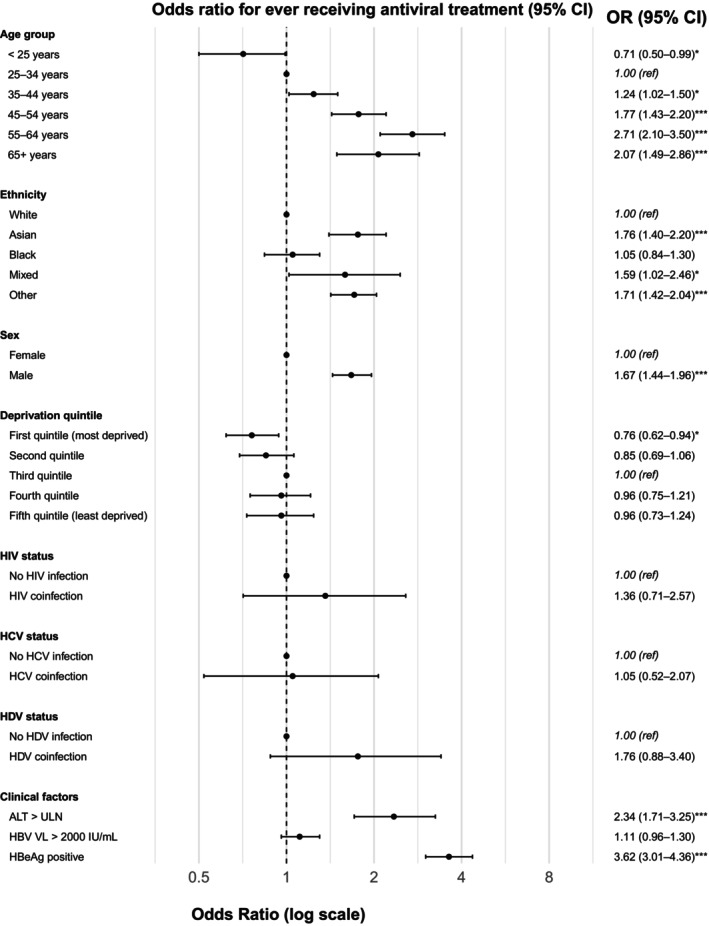
Forest plot showing factors associated with NA antiviral treatment in adults living with chronic Hepatitis B (CHB) in the UK. Main multivariable logistic regression model from analysis undertaken in the Health Informatics Collaborative secondary care electronic health record database (*n* = 7558). *ALT > ULN defined as ≥ 30 U/L in males and ≥ 19 U/L in females as per WHO guidelines [12]. † First IMD quintile denotes most deprived quintile, fifth quintile denotes least deprived. †† Measured at any point throughout entire study duration. OR, odds ratio; IMD, index of multiple deprivation; HIV, human immunodeficiency virus; HCV, hepatitis C virus; HDV, hepatitis Delta virus.

A minority of the population had a record of NA treatment during the period of follow‐up (*n* = 2014/7558, 26.6%; Table 2). In the multivariable model, HBeAg positivity (OR 3.62, 95% CI 3.01–4.36) and ALT> ULN (OR 2.34, 95% CI 1.71–3.25) were associated with NA prescription (Table 3, Figure 1), in line with clinical indications for starting treatment [6]. Male sex (OR 1.67, 95% CI 1.44–1.96) and increasing age at first record of CHB status (ORs for ages 45–54 years, 55–64 years, and ≥ 65 years age 1.77 (95% CI 1.43–2.20), 2.71 (95% CI 2.10–3.50) and 2.07 (95% CI 1.49–2.86), respectively) were also statistically associated with treatment (Table 3, Figure 1). Individuals belonging to Asian and ‘Other’ ethnicities were more likely to be treated (ORs 1.76 [95% CI 1.40–2.20] and 1.71 [95% CI 1.42–2.04], respectively), compared to individuals of White ethnicity (Table 3, Figure 1). Belonging to the first IMD quintile (most deprived) was associated with a 24% reduction in the odds of NA treatment (OR 0.76, 95% CI 0.62–0.94) as compared to the third quintile.

**TABLE 3:** Logistic regression models investigating factors which are associated with antiviral treatment in adults with CHB.

Characteristic	Univariable OR (95% CI)	Multivariable OR (95% CI)
Age group		
< 25 years	1.02 (0.79–1.31)	0.71 (0.50–0.99)*
25–34 years	1.00 (ref)	1.00 (ref)
35–44 years	1.20 (1.04–1.39)*	1.24 (1.02–1.50)*
45–54 years	1.92 (1.65–2.24)***	1.77 (1.43–2.20)***
55–64 years	2.21 (1.86–2.63)***	2.71 (2.10–3.50)***
≥ 65 years	1.82 (1.48–2.24)***	2.07 (1.49–2.86)***
Ethnicity (self‐reported)		
White	1.00 (ref)	1.00 (ref)
Asian	1.67 (1.42–1.96)***	1.76 (1.40–2.20)***
Black	1.08 (0.93–1.25)	1.05 (0.84–1.30)
Mixed	1.25 (0.9–1.72)	1.59 (1.02–2.46)*
Other	1.70 (1.49–1.93)***	1.71 (1.42–2.04)***
Sex		
Female	1.00 (ref)	1.00 (ref)
Male	1.66 (1.49–1.84)***	1.67 (1.44–1.96)***
Index of multiple deprivation (IMD) quintile		
First quintile (most deprived)	0.85 (0.73–1.00)	0.76 (0.62–0.94)*
Second quintile	0.87 (0.74–1.03)	0.85 (0.69–1.06)
Third quintile	1.00 (ref)	1.00 (ref)
Fourth quintile	0.99 (0.83–1.17)	0.96 (0.75–1.21)
Fifth quintile (least deprived)	1.03 (0.85–1.24)	0.96 (0.73–1.24)
Coinfection status		
No HIV infection	1.00 (ref)	1.00 (ref)
HIV coinfection	2.18 (1.43−3.31)***	1.36 (0.71–2.57)
No HCV infection	1.00 (ref)	1.00 (ref)
HCV coinfection	1.52 (0.94–2.40)	1.05 (0.52–2.07)
No HDV infection	1.00 (ref)	1.00 (ref)
HDV coinfection	1.75 (1.08–2.77)*	1.76 (0.88–3.40)
Laboratory markers		
ALT > ULN^a^, ^b^	2.03 (1.65–2.52)***	2.34 (1.71–3.25)***
HBV VL > 2000 IU/mL^b^	1.37 (1.22–1.53)***	1.11 (0.96–1.30)
HBeAg positive^b^	2.80 (2.43–3.22)***	3.62 (3.01–4.36)***

Abbreviations: ALT, alanine aminotransferase; CT, computed tomography; HBeAg, hepatitis B virus E antigen; HBV VL, hepatitis B virus viral load; HCV, hepatitis C virus; HDV, hepatitis Delta virus; HIV, human immunodeficiency virus.

* *p* < 0.05.

*** *p* < 0.001.

^a^ ALT ULN (upper limit of normal) ≥ 30 U/L in males and ≥ 19 U/L in females.

^b^ Mixed‐effects logistic regression model, with unique individual identifier fitted as a random effect.

### 3.3 Influence of Changing Guidelines on Proportion of Population Reaching Treatment Eligibility Criteria

**FIGURE 2:**
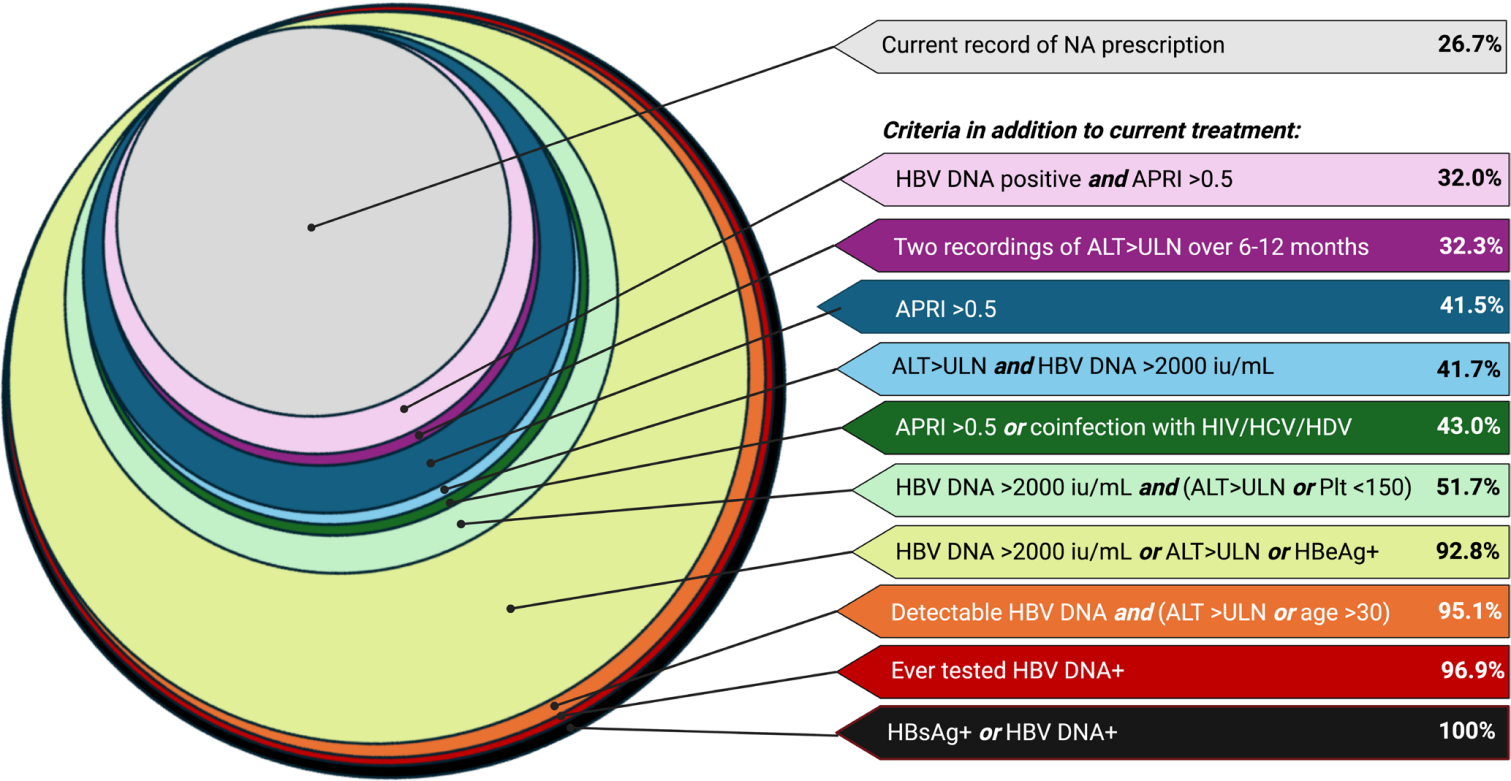
Visualisation of the impact of relaxing treatment criteria on the proportion of adults with CHB eligible for antiviral treatment with NA agents. We first calculated the proportion of the current population under treatment and then considered extended treatment scenarios, calculating the total currently on treatment plus those additionally meeting the relaxed treatment criteria. These data are also presented in tabulated form in Table S1. APRI—aspartate aminotransferase to platelet ratio index = [(AST/upper limit of normal) × 100/platelet count], with AST ULN = 40 IU/L. ALT (alanine transaminase) ULN = upper limit of normal, defined by WHO guidelines as 30 U/L for males and 19 U/L for females. HIV, human immunodeficiency virus; HCV, hepatitis C virus, HDV, hepatitis D virus. HBsAg, hepatitis B surface antigen. HBV DNA indicates viral load (VL) in IU/mL. Platelet count (Plt) x10^9^/L.

Having calculated the proportion of our clinical population with a record of antiviral treatment to date, we expanded this to reflect potential relaxation of treatment criteria (Figure 2, Table S1).

- Observing the real‐world cohort (in which therapy has been previously informed by UK or EASL guidelines [6, 10] alongside patient and prescriber preference), 26.7% (2014/7558) of individuals had a record of NA treatment; this is the baseline real‐world population on treatment.
- Considering treatment for those testing HBV DNA positive and with recorded APRI > 0.5 would expand the pool offered treatment by 406 individuals (to a total of 2420/7558); thus overall 32.0% would be offered treatment based on this EASL recommendation.
- Applying the WHO treatment criterion of two ALT measurements > ULN during any 6–12‐month period, (regardless of other markers) an additional 726 individuals would have met treatment criteria during the period observed; thus overall 32.3% (2740/7558) of the observed population would be offered treatment.
- Based on an APRI score > 0.5 as a treatment indicator in line with new WHO guidance [12], irrespective of other markers, an additional 1123 individuals would have been considered treatment eligible, such that 41.5% (3137/7558) of the observed population would be offered treatment.
- Applying laboratory thresholds of HBV VL > 2000 IU/mL *and* ALT > ULN, in accordance with new WHO guidance [12] (and also concordant with Brazilian national guidance [14, 15]), an additional 1138 individuals would meet criteria, such that 41.7% (3152/7558) of the population would be offered treatment.
- If accounting for APRI > 0.5 or the additional indication of HIV/HCV/HDV coinfection, an additional 111 individuals become eligible, taking the total treated population to 43.0% (3248/7558).
- By combining HBV VL > 2000 IU/mL with either ALT> ULN or a low platelet count, 3908/7558 individuals would become treatment eligible, with 51.7% of the population then being offered treatment.
- Based on expanded EASL criteria, to treat if any of the following (at any timepoint) HBV VL > 2000 *or* ALT> ULN *or* HBeAg+, we identified an additional 5002 who would become treatment eligible, taking the total on treatment to 92.8% (7016/7558).
- By applying recent Chinese treatment recommendations [13], which suggest offering treatment to all those with a detectable HBV VL with either ALT > ULN or age > 30 years, 95.1% (*n* = 7187/7887) of individuals would potentially be treated;
- Using the most relaxed EASL criterion, any positive test for HBV DNA at any time point, the number to be offered treatment would increase to 96.9% (7325/7558);
- To provide context for the ‘treat all’ paradigm, we also consider application of the most relaxed threshold, namely offering treatment to those with any marker of HBV infection (HBsAg and/or HBV DNA positivity), irrespective of quantification of other markers. By definition, this would make 100% of the population eligible for treatment.

## 4 Discussion

### 4.1 Antiviral Treatment in CHB—Current and Future Eligibility

The HIC database is the largest secondary care data source containing longitudinal laboratory parameters for viral hepatitis in the UK and representing real‐world clinical data collected from individuals who are accessing health services at multiple NHS Trusts across the country, with increasing potential for generalisation as the database expands. In this preliminary descriptive analysis of adults with CHB accessing specialist secondary/tertiary care services across six centres in England, as expected, < 30% had a record of NA treatment, reflecting recommendations in both UK/European [6, 10] and global guidelines prior to 2024 [9]. This treated proportion is comparable to that reported from other cohorts elsewhere in the world [22, 23, 24]. As treatment criteria are progressively relaxed, it is recognised that an increasing proportion of the population living with HBV will become treatment eligible, but the expanded population eligible for treatment has not yet been carefully enumerated in different settings.

This analysis of selected sites in England suggests that ~30%–50% will meet the new laboratory thresholds that are likely to be proposed in most settings (scenarios (ii)‐(vi) in results), with the potential for expansion to 80%–100% (scenarios (vii)‐(xi)) if the most relaxed criteria are applied. This projection provides preliminary estimates that are relevant to planning service delivery and resource allocation. Future analysis is warranted to investigate potential differences in data collection timeframes, sample sizes, and treatment patterns, and individual patient/prescriber preference across sites. While these factors are outside of the scope of the present analysis, further investigation is planned to quantify possible variations. Potential differences across English sites may indicate associations of NHS Trust‐ and/or regional‐level factors with clinical practice and implementation of clinical guidelines, which are diluted when data from all sites are pooled.

### 4.2 Benefits and Risks of Expanding Therapy

Previous global HBV recommendations have been influenced by the availability and affordability of drug therapy, potentially reflecting a need to ration resources in some settings. However, as safety, accessibility, and affordability have improved (largely due to the widespread availability of generic NA preparations) [6], eligibility criteria are being expanded to improve outcomes and reduce health inequities. Older guidelines, which inform prescribing practice to date, are based on large historical cohorts, often observing specific populations in which parameters at baseline were used to estimate the longer‐term risk of liver‐related events (with a lack of high‐quality longitudinal off‐treatment data).

Expansion of treatment coverage would benefit individuals through the reduction of the risk of progression to end‐stage disease, and bring public health benefits through reducing transmission and offering overall cost benefits [25, 26]. Thus, treatment expansion supports progression towards global 2030 elimination targets [5, 27]. However, the specific risks and benefits of treatment expansion vary between settings, and careful evaluation is required to assess optimum interventions in different populations.

### 4.3 Advances Towards Global Elimination Targets

The UK Health Security Agency (UKHSA) has highlighted areas in which enhanced efforts are required to attain elimination targets, which include enhanced interventions and resources to provide treatment, as well as the need for improved data collection. A hepatitis B ‘dashboard’ is proposed to visualize and assess testing uptake, diagnostic results, and treatment, incorporating characteristics groups such as age, sex, ethnicity, country of birth, and deprivation, and therefore to identify gaps, risk groups, and opportunities for intervention (https://www.gov.uk/government/publications/hepatitis‐b‐in‐england/hepatitis‐b‐in‐england‐2024). Our approach makes advances in this direction, albeit only for the limited population represented in the HIC dataset.

### 4.4 HBV Treatment and Health Inequity

Belonging to the first IMD quintile (most deprived) was associated with a significant reduction in the odds of antiviral treatment, even in this study which by definition only includes those already linked to care and under follow‐up. The true equity gap is therefore likely to be higher than estimated here. This result highlights a need for strategies to reduce health inequity and to ensure that vulnerable and marginalised groups are offered treatment, provided with education, and are supported to access therapy when it is indicated. People living with HBV infection who are not engaged in long‐term follow‐up and treatment may have further specific vulnerabilities [28] and potentially worse outcomes. For example, vulnerable migrants and refugees suffer specific challenges (high mobility, language barriers, stigma, competing health and psychosocial challenges) which are known to be barriers to access and affordability of healthcare [28, 29].

### 4.5 Factors Associated With Treatment Eligibility

Although we found a relationship between treatment and HBeAg status and ALT > ULN, there was not a statistical relationship between HBV VL and receipt of treatment. This lack of association can be explained, as those who entered the cohort already on treatment will typically have suppressed VL throughout. We considered additional analysis to determine the relationship between VL at baseline and at treatment start date, but retrospective data (especially dates of starting treatment) are not reliably captured in electronic datasets, and the majority of individuals with a record of treatment are already virologically suppressed at their first timepoint recorded (60% in a recent analysis of the same population [30]). Uncertainty around treatment start dates also precludes detailed analysis of transition from untreated to treated status.

We expected to find an association between HIV status and receipt of treatment, since all those living with HIV should be treated, and regimens overlap with HBV therapy. However, the number in this group is small and we recognize some treatment data are missing for the HIV/HBV coinfection group in the HIC dataset, as a result of differences in the way HIV care is delivered: when antiviral prescriptions are issued through Sexual Health services, anonymization of clinical records prevents complete data linkage (accounting for an underestimate in the proportion of the HIV/HBV population prescribed treatment).

The association between ethnicity and antiviral treatment initiation is likely to be influenced by unmeasured host and viral characteristics, including viral genotypes which cluster in different populations according to country of birth. Although genotype is not determined in routine clinical practice, it can nevertheless influence the phenotype of infection—for example, influencing VL and/or HBeAg status and therefore influencing treatment eligibility. The CHB population in the United Kingdom is disproportionately socioeconomically deprived [31], and it is likely that IMD quintile associates with diverse other factors that influence treatment access and eligibility. Further characterisation of the social and economic characteristics of the UK CHB population is warranted, with a need for special scrutiny of groups who are under‐represented as a result of systematic barriers to care.

### 4.6 Stopping Therapy

In any of the scenarios we evaluated, not all individuals offered antiviral therapy would wish to take it, and a small proportion may clear HBsAg (either in the context of acute or chronic infection), allowing therapy to be stopped [32]. Treatment may also be stopped if prophylactic NA therapy is administered for a defined period peri‐partum to prevent vertical transmission [33]. Although this small minority may stop therapy, this does not influence the overall research question addressed here, and for the purpose of this analysis, we assumed that adults who started NA treatment in the time period observed will have continued long‐term.

### 4.7 Caveats and Limitations

The data presented here represent only six large, urban centres in England; trends may differ across settings and additional data will be required to determine the extent to which our observations apply in other regions of the United Kingdom, in other high‐income settings, and in low/middle‐income economies [28, 29]. Our analysis is biased by only capturing data for individuals who are diagnosed, linked to care, and attending follow‐up in specified centres. Quantifying the impact of this bias is difficult, but missing diagnoses and linkage in vulnerable groups may mean that we underestimate overall treatment eligibility.

The nature of the electronic records we have accessed makes a record of a treatment prescription being issued by a healthcare provider, which does not always correlate with the collection of medication, or subsequent adherence to daily therapy. Continuity and treatment breaks are not areas that have been well assessed and require further research.

The HIC framework collects a subset of data, but does not systematically record other parameters that may influence treatment prescription such as comorbidity (e.g., metabolic liver disease, diabetes, immunosuppression), extrahepatic complications (e.g., glomerulonephritis, vasculitis) or family history (Table S2). Most of these groups should be incorporated in those already on treatment at baseline (in keeping with older guidelines), but these important unmeasured factors may bias the observed associations and could lead to an underestimation of the total population eligible for treatment.

As elastography scores have not been consistently recorded in different EPR systems, there is a high level of missingness (Table S3) and we were not able to investigate the association between elastography scores and treatment eligibility in this study. While an alternative measure of liver fibrosis can be ascertained by using an APRI score, this approach is also limited by missingness (Table S4), as AST is often not measured as part of the routine panel of liver biochemistry. We elected not to impute fibrosis scores given the high percentage of missingness. Due to these missing parameters, our dataset underestimates treatment based on liver fibrosis; however, many of these individuals may either already be on treatment or meet an alternative eligibility criterion (e.g., based on VL or ALT).

We did not analyse any quantitative HBsAg data because this was not measured consistently between sites (some laboratories only report a categorical measurement, while others undertake semi‐quantitative or fully quantitative measurements) and clinical practice varies (in some individuals it would be measured only at baseline, while others repeat the measurement at varying time intervals).

Although we have the advantage of a diverse cohort representing a range of ethnicities (and therefore viral genotypes), the proportions of individuals who are treatment eligible will vary between settings, and further data are needed to represent other populations. Ethnicity is broadly captured in EHR, but country of birth is not typically recorded (the latter may be more relevant as it is more likely to reflect the risk, route and age of HBV acquisition, and HBV genotype). The relationship between treatment eligibility and people reported as being in ‘other’ ethnic groups needs further scrutiny to understand the characteristics of individuals represented in this broad and non‐specific category. While we are not able to fully explore the reasons for different rates of treatment according to ethnicity, we speculate that there may be biological differences that are unmeasured in this dataset (e.g., host genetics and viral genotype) that influence the chance of meeting treatment eligibility criteria. There are also a wide number of other socio‐demographic, environmental and clinical parameters that could influence the likelihood of meeting treatment criteria or being offered or accepting treatment, which are not captured in this study.

### 4.8 Future Aspirations

Prospectively, the HIC is setting out to focus more analysis on clinically important endpoints, such as fibrosis, cirrhosis and hepatocellular carcinoma (HCC), which will allow us to evaluate (i) these outcome markers as determinants of treatment eligibility (patients in these groups should already be offered treatment) and (ii) how changes in prescribing practice influence the evolution of these outcomes. An updated dictionary for data collection now includes ICD‐10 coding for endpoints, which will allow us to better capture pathology including cirrhosis, HCC, and non‐hepatic comorbidities, together with approaches for collecting data from free‐text imaging reports using Natural Language Processing and AI techniques. At the time of this analysis, fibroscan data were not reliably recorded and ICD‐10 codes were not available for participating sites, although proof‐of‐principle has been established [34]. Quantification of HBsAg is an additional variable that can potentially influence treatment eligibility and should be added to future analyses as it becomes more consistently measured in routine clinical practice in the UK.

### 4.9 Global Landscape for Treatment Expansion

It is likely that further national and international guidelines will broadly follow the precedents set by the WHO, EASL, Brazil and China, based on consensus across expert bodies [35, 36]. However, the specific recommendations, thresholds and nuance of guidelines will undoubtedly remain—to some extent—setting dependent. For example, the ‘conditional’ recommendations for wide treatment of all who test HBsAg‐positive set out by the WHO may be crucial for the roll‐out of equitable treatment in settings where resources are limited, but are unlikely to be relevant in most high‐income countries, especially with state‐funded healthcare provision, where there is reliable access to tools for risk stratification (VL, liver enzymes, elastography).

While TDF and ETV are broadly efficacious, cheap, and well tolerated with minimal toxicity [6], prescription of these agents nevertheless requires access to diagnostics, laboratory testing, imaging, and a secure supply of affordable drugs. In regions where access to health infrastructure and laboratory stratification is challenging, the simplest assessments are required, including the option to offer treatment to any adult who tests HBsAg‐positive. While HBV DNA assessment is widely available across the UK (and there are no additional costs to the patient to access this test), we have nevertheless applied WHO guidelines that account for situations in which this is not available in order to inform understanding of the potential impact of this simplified criterion.

Evaluation of demands on health service infrastructure is key to ensuring that treatment expansion can be implemented in the real world. In different settings, change in guidelines may accelerate decentralisation of HBV management to primary care, integration of HBV care with other services, and investment in triple elimination programmes that tackle HBV together with HIV and syphilis. Cost‐effectiveness analysis of different models will be needed, supported by close partnerships between policymakers, clinical services and the pharmaceutical industry to support access to HBV therapy.

WHO guidelines now include treatment for adolescents (with those aged ≥ 12 years being treatment‐eligible under the same criteria as adults). This group is outside the scope of this paper as HIC data only include adults, but representation of younger people in research and clinical data will be crucial to understand implications for the delivery of treatment.

Our analysis highlights that health inequity represents a barrier to HBV treatment, and implementation strategies need to focus on the provision of accessible, acceptable, and affordable pathways to clinical services, which recognize and redress social, economic, cultural and linguistic barriers and challenges, and offer equitable testing and treatment. Future scale‐up of successful long‐term treatment will rely not only on the simplification of treatment eligibility criteria but also on a wide range of service improvements including better access to culturally relevant information, education of healthcare providers, decentralization of care, provision of HBV services linked to other clinical programs (liver health, mental health, family services) and provision of peer support.

### 4.10 Patient Representation

Patient voice and involvement of the community as stakeholders has increasingly been recognised as an important driver of guidance, recognising that individual preference is a fundamental component of therapeutic decision making [37, 38], and with civil society organisations recognised as essential contributors to clinical guidelines. In many instances, those outside strict treatment criteria may express a wish to receive treatment, while in other cases those who do meet clinical thresholds for treatment may choose not to embark on what is currently life‐long therapy.

## 5 Conclusions

We have quantified treatment coverage across a large population of adults with CHB, demonstrating that a minority of individuals currently receive antiviral treatment but predicting the pattern of increasing eligibility in line with changing eligibility criteria. Further analyses incorporating larger datasets and tackling data missingness will be possible as the HIC dataset expands. Such data are crucial to provide evidence to inform the resourcing, infrastructure, and implementation of treatment programmes, and to tackle health inequities, thus supporting progress towards elimination targets.

## Conflicts of Interest

Cori Campbell received doctoral funding from GSK. Eleanor Barnes and Philippa C. Matthews have academic collaborative partnerships with GSK.

## Supporting information


**Table S1:** Table to show the impact of relaxing antiviral treatment initiation criteria on the number and proportion of individuals eligible for antiviral treatment.
**Table S2:** Clinical and sociodemographic criteria recommended to inform for HBV treatment by EASL guidelines, which are not captured in laboratory or imaging data.
**Table S3:** Types of hepatic imaging performed in individuals included in analysis of Health Informatics Collaborative data, investigating factors associated with antiviral treatment initiation in chronic HBV infection.
**Table S4:** Frequency of missing laboratory parameters/scores in HIC dataset evaluated for HBV treatment eligibility.

## Data Availability

The NIHR HIC Data Sharing Framework provides an overarching governance approach for data sharing between NHS organisations for research purposes. Research proposals are reviewed by the NIHR HIC Viral Hepatitis and Liver Disease theme Scientific Steering Committee. The governance terms of the HIC pertaining to clinical data for the theme do not allow clinical data to be shared outside the Trusted Research Environment hosted by Oxford University Hospitals NHS Foundation Trust. More information about access to datasets within NIHR HIC data collaborations can be found on our website: https://hic.nihr.ac.uk/.
