# Supplementary material for: Hepatitis B Virus (HBV) Treatment Eligibility in the UK: Retrospective Longitudinal Cohort Data to Explore the Impact of Changes in Clinical Guidelines
Source: J Viral Hepat. 2025 Oct 23;32(11):e70098. doi: 10.1111/jvh.70098 (PMC12548009; doi:10.1111/jvh.70098)
Supplement: Supplementary file 1 — Table S1: Table to show the impact of relaxing antiviral treatment initiation criteria on the number and proportion of individuals eligible for antiviral treatment. Table S2: Clinical and sociodemographic criteria recommended to inform for HBV treatment by EASL guidelines, which are not captured in laboratory or imaging data. Table S3: Types of hepatic imaging performed in individuals included in analysis of Health Informatics Collaborative data, investigating factors associated with antiviral treatment initiation in chronic HBV infection. Table S4: Frequency of missing laboratory parameters/scores in HIC dataset evaluated for HBV treatment eligibility. [file JVH-32-0-s001.pdf]

SUPPLEMENTARY MATERIAL

Hepatitis B Virus (HBV) treatment eligibility in the UK: retrospective longitudinal cohort data to explore the impact of changes in clinical guidelines

CONTENTS OF SUPPLEMENTARY FILE

Table S1. Table to show the impact of relaxing antiviral treatment initiation criteria on the number and proportion of individuals eligible for antiviral treatment.

Table S2: Clinical and sociodemographic criteria recommended to inform for HBV treatment by EASL guidelines, which are not captured in laboratory or imaging data.

Table S3: Types of hepatic imaging performed in individuals included in analysis of Health Informatics Collaborative data, investigating factors associated with antiviral treatment initiation in chronic HBV infection.

Table S4: Frequency of missing laboratory parameters/scores in HIC dataset evaluated for HBV treatment eligibility

Table S1. Table to show the impact of relaxing antiviral treatment initiation criteria on the number and proportion of individuals eligible for antiviral treatment. Different scenarios were considered using data from the Health Informatics Collaborative (representing England) (10), in each case building on the population already receiving treatment based on expansion of eligibility criteria.

**Scenario**	**Total, n (%)**
(i) Currently receiving NA therapy, n (%)	2014/7558 (26.7%)
(ii) Scenario (i) ***and*** HBV DNA positive ***and*** APRI >0.5	2420/7558 (32.0%)
(iii) Scenario (i) ***and/or*** two ALT > ULN[b] during a 6-12-month period, regardless of other markers, n (%)	2740/7558 (32.3%)
(iv) Scenario (i) ***and/or*** APRI[a] >0.5	3137/7558 (41.5%)
(v) Scenario (i) ***and/or*** VL >2000 IU/ml ***and*** ALT >ULN	3152/7558 (41.7%)
(vi) Scenario (i) ***and/or***<br>• APRI > 0.5<br>***or***<br>• Coinfected with HIV, HCV and/or HDV	3248/7558 (43.0%)
(vii) Scenario (i) ***and/or***<br>• VL > 2000 IU/ml ***and*** ALT >ULN<br>***OR***<br>• Plt <150x10^9/ml	3908/7558 (51.7%)
(viii) Scenario (i) ***and/or***<br>• VL >2000<br>***or***<br>• ALT>ULN<br>***or***<br>• HBeAg+	7016/7558 (92.8%)
(ix) Scenario (i) ***and/or***<br>• Detectable VL AND:<br>• ALT > ULN[b]<br>***or***<br>• Age > 30 years	7187/7558 (95.1%)
(x) Scenario (i) ***and/or*** any positive test for HBV DNA at any time point	7325/7558 (96.9%)
(xi) HBsAg ***and/or*** HBV DNA positivity (i.e. treatment of all individuals with markers of HBV infection)	7558/7558 (100%)

NA, nucleos/tide analogue; ALT, alanine aminotransferase; VL, viral load; HBsAg, hepatitis B virus surface antigen, > ULN - Above the upper limit of normal (defined as ≥30 IU/ML in males and ≥19 IU/ML in females).
[a] APRI – aspartate aminotransferase to platelet ratio index = (AST/AST upper limit of normal) X 100/platelet count x10^{9}/L], with AST ULN = 40 IU/L.
[b] ULN for ALT defined by WHO guidelines as 30 U/L for males and 19 U/L for females.

Table S2: Clinical and sociodemographic criteria recommended to inform for HBV treatment by EASL guidelines, which are not captured in laboratory or imaging data. These parameters are not recorded in the HIC dataset and therefore are not assessed as part of the current approach to quantifying treatment eligibility.

**Criterion**	**Application to treatment eligibility**
Family history	Family history of HCC
Location of birth	Lower threshold for treatment in those born in Africa / Oceania
Smoking and alcohol	Assess smoking history and alcohol (especially with intake $\geq$60 g/day*)
Metabolic risk factors	Body mass index $\geq$30<br>$\geq$3 metabolic factors (overweight/obesity, hypertension, dyslipidaemia, diabetes, hepatic steatosis)
Extrahepatic complications of HBV infection	E.g. glomerulonephritis, vasculitis, panarteritis nodosa
Immunosuppression	Lower threshold for treatment in individuals with immunosuppressive condition or medication
Risk for HBV transmission	Assessed from history (context, behaviour, likelihood/type/frequency of exposure events)

HCC – hepatocellular carcinoma,
* Alcohol intake equivalent to 6 standard alcoholic drinks

Table S3: Types of hepatic imaging performed in individuals included in analysis of Health Informatics Collaborative data, investigating factors associated with antiviral treatment initiation in chronic HBV infection. Data are presented from 3 NHS trusts contributing imaging data both overall (n = 2665) and stratified by those who initiated antiviral treatment (n = 547) and did not initiate treatment (2118) throughout follow-up. Percentages are expressed out of the column total.

**Characteristic**	**Total, n (%)**	**Untreated, n (%)**	**Treated, n (%)**
N	2665	2118	547
Elastography, n (%)	355 (13.3)	336 (15.9)	19 (3.5)
Liver stiffness score, mean (SD)	6.23 (5.28)	6.11 (5.21)	8.33 (6.13)
MRI, n (%)	65 (2.4)	28 (1.3)	37 (6.8)
CT, n (%)	106 (4.0)	61 (2.9)	45 (8.2)
Ultrasound, n (%)	1018 (38.2)	632 (29.8)	386 (70.6)

SD, standard deviation; MRI, magnetic resonance imaging; CT, computed tomography.

Table S4: Frequency of missing laboratory parameters/scores in HIC dataset evaluated for HBV treatment eligibility

**Parameter**	**Number (%) missing value**
HBV Viral Load	173 (2.3%)
Platelet count	246 (3.3%)
HBeAg status	1606 (21.2%)
APRI score	2785 (36.8%)
